# CMOS Detector Staggered Array Module for Sub-Terahertz Imaging on Conveyor Belt System

**DOI:** 10.3390/s23031232

**Published:** 2023-01-20

**Authors:** Moon-Jeong Lee, Ha-Neul Lee, Ga-Eun Lee, Seong-Tae Han, Dong-Woo Kang, Jong-Ryul Yang

**Affiliations:** 1 Department of Electronic Engineering, Yeungnam University, Gyeongsan 38541, Republic of Korea; 2 Electrophysics Research Center, Korea Electrotechnology Research Institute, Changwon 51543, Republic of Korea; 3 AI Compact SoC Research Section, Electronics and Telecommunications Research Institute, Daejeon 34129, Republic of Korea

**Keywords:** array layout, CMOS detector, conveyor belt, high-resolution, replaceable array, sub-terahertz imaging

## Abstract

A complementary metal–oxide–semiconductor (CMOS) detector array is proposed to improve the sub-terahertz imaging resolution for objects in the conveyor belt system. The image resolution is limited to the implemented configuration, such as the wide spacing in the detector array, the high conveyor belt speed, and the slow response of the signal conditioning block. The proposed array can improve the image resolution in the direction perpendicular to the movement of the belt, which is determined by the size and interval of the detector pixel, by configuring the array into two replaceable columns located at the misaligned horizontal positions. Replaceable detector unit pixels are individually attached to the motherboard after measuring and evaluating the detection performance to construct the proposed array. The intensities of 32 detector pixels placed under the conveyor belt with a width of 160 mm were initially calibrated in every image, including the beam pattern of 0.2 THz signals generated from the gyrotron. The image resolution of the perpendicular direction obtained from the proposed array was measured to be approximately 5 mm at a conveyor belt speed of 16 mm/s, demonstrating a 200% improvement in resolution compared to the conventional linear array under the same conditions.

## 1. Introduction

Electromagnetic (EM)-wave imaging, based on the transmission and reflection characteristics of wireless signals is a valuable technology for applications such as foreign body detection, security gate checks, and microcrack/void detection [1,2,3]. EM-wave imaging shows the dielectric properties and surface conditions, providing non-homogeneous characteristics of the object mediums that are difficult to obtain using human vision [4]. The resolution of EM-wave imaging is generally determined by the wavelength of the transmitted and received signals [5]. Sub-terahertz imaging using millimeter-wave signals of 100 GHz or higher is particularly promising in conveyor belt systems to detect foreign bodies in food and unwanted voids in products owing to its low ionization energy transfer and high resolution [6].

The sub-terahertz imaging system for obtaining transmitted images of objects on a conveyor belt consists of a transmitting block that radiates sub-terahertz signals, optical components that help the signals to be evenly applied to the objects on the belt, a receiving block that captures the signal intensity, and signal conditioning circuits that amplify and filter the data for real-time imaging [7]. It is necessary to implement a high-power transmitter and a highly sensitive detector to improve the image quality, because the signals are scattered by the objects and the belt [8]. The spatial resolution of the sub-terahertz imaging system is determined by the detector element density per unit area [9]. Furthermore, the operating frequency determines the spatial resolution, because the number of detectors implemented per unit area depends on the antenna size for capturing the signals. Thus, the general method for improving the resolution of sub-terahertz imaging is by increasing the operating frequency [10,11,12]; however, this can deteriorate the signal-to-noise ratio (SNR) of the obtained image owing to the lower transmitting power and receiving sensitivity and can also increase the system implementation cost. Methods for arranging more detector elements per unit area by reducing the physical size for antenna implementation have been proposed by introducing antenna shapes, additional stacked structures, and artificial magnetic conductors [13,14,15]. An increase in the detector elements owing to a decrease in an antenna size may affect the detection performance degradation owing to the crosstalk between adjacent antennas [16]. Techniques for using phase characteristics, broadband transmitters and receivers, or heterodyne configurations have been proposed to increase the image resolution and quality; however, there is a limitation in obtaining a large-scale image, because the resolution is only enhanced in a small area, the location of the target is fixed, or the complexity of the implementation is increased [17,18,19]. Techniques for increasing the image resolution of terahertz spectroscopy have been proposed using the correlation from adjacent pixels in the detector array; however, these are not suitable for sub-terahertz real-time imaging, because a complex mathematical process is required for the implementation [20,21,22]. In the resolution enhancement technique using the correlation of adjacent pixels, an image error can significantly increase when there is a malfunction or bad pixel in the sub-terahertz detector array.

In this study, a detector array structure is proposed to increase the spatial resolution of the sub-terahertz imaging of a target on a conveyor belt. The proposed detector array consists of two lines of the detector array that are intentionally misaligned and have a configuration in which the detectors in the second column are placed between the detectors in the first column. The complementary metal–oxide–semiconductor (CMOS) detector integrated circuit (IC), including an on-chip antenna, detector core, preamplifier, and buffer amplifier, is attached to a unit detector module. The proposed array configuration is implemented by connecting a unit module to a single motherboard, on which the unit module can be replaced or modified when the detector IC on the unit module malfunctions or the responsivity of the module is significantly different from that of the other modules. Section 2 presents the operating principle of the proposed array configuration for increasing the spatial image resolution on a conveyor belt. The implementation of both the CMOS detector IC with the on-chip antenna and the proposed array is presented in Section 3. Section 4 provides the measurement results of the voltage responsivities of the CMOS detector ICs in the array and the images of the object on the conveyor belt obtained by the proposed array. The improvement in the proposed array is discussed in Section 4. The concluding remarks are presented in Section 5.

## 2. Proposed CMOS Detector Array for Conveyor Belt System

### 2.1. Full-Wave Imaging on the Conveyor Belt

**Figure 1:**
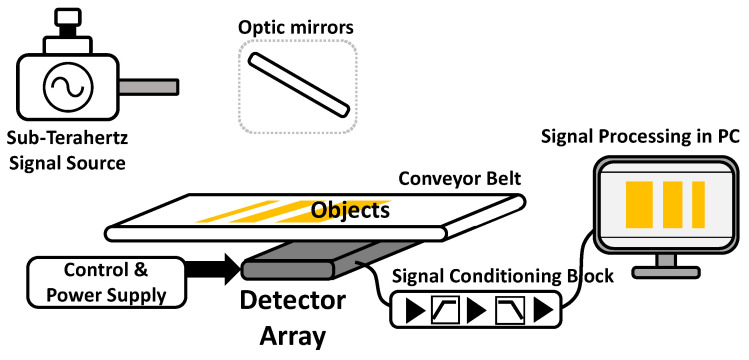
Sub-terahertz full-wave imaging system for the target objects on a conveyor belt.

Figure 1 presents a block diagram of the sub-terahertz full-wave imaging system for an object placed on a conveyor belt. The sub-terahertz signals continuously generated from the signal generator pass through an optical system that can irradiate the entire width of the conveyor belt and are evenly incident in the direction perpendicular to the moving direction of the conveyor belt. The signals passing through the object and conveyor belt are transmitted to the detector array mounted inside the conveyor belt. The power level of the transmitted signal depends on the material, uniformity, homogeneity, layer stacks, and other characteristics of the object. The sub-terahertz image can be obtained from the detected output voltages owing to these relative characteristic differences. The transmitting signal is generally fixed at a narrow frequency, and the detectors in the array only collect the signal at the same narrow frequency. A frequency of 0.2 THz is selected in this study for the detection of foreign bodies in food owing to the large difference in the absorption characteristics by water and humidity [23,24,25]. After being amplified and filtered in the signal conditioning block, the outputs of the detector array are reconstructed to the image during signal processing, considering the detector pixel position and the movement of the conveyor belt. The mechanical or electrical modulation technique, which moves the output signal from a direct current (DC) to an arbitrary frequency, can be used in imaging systems to avoid deterioration by the flicker noise of the detector [7].

### 2.2. Operating Principle of the Proposed Array

Assuming that sub-terahertz waves with a constant magnitude are irradiated onto the conveyor belt in the shape of a linear beam, the spatial resolution of the sub-terahertz imaging system for objects on the conveyor belt is determined by the arrangement of the detector array and number of detector arrays per unit area. When configuring the detector array without an optical lens, it is advantageous for a high spatial resolution to increase the number of power detectors per unit area. However, increasing the number of detectors per unit area has limitations owing to the physical dimensions of the integrated antenna in the detector, the implementation of interface circuits for control and power supply, and the interference between the adjacent detectors in an array. Integrating several detectors in a single chip is an effective method for increasing the spatial resolution; however, this can lower the resolution of the image when the detector malfunctions or has a significantly different response compared to the others. 

**Figure 2:**
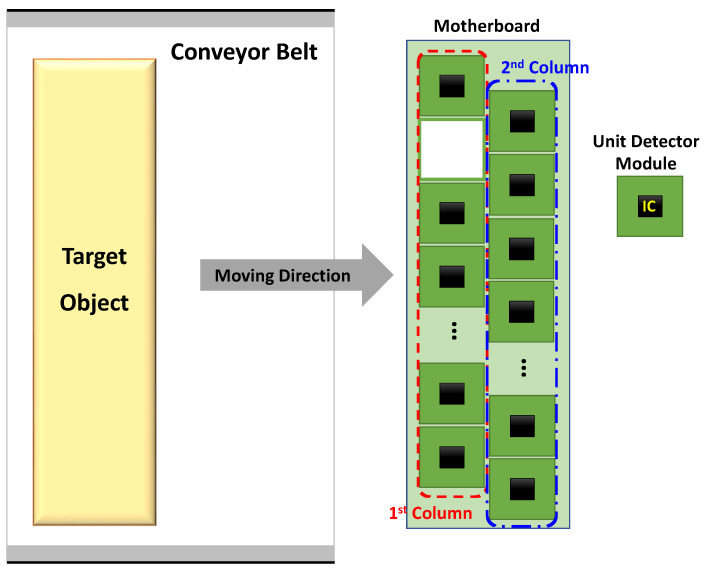
Configuration diagram of the proposed detector array.

**Figure 3:**
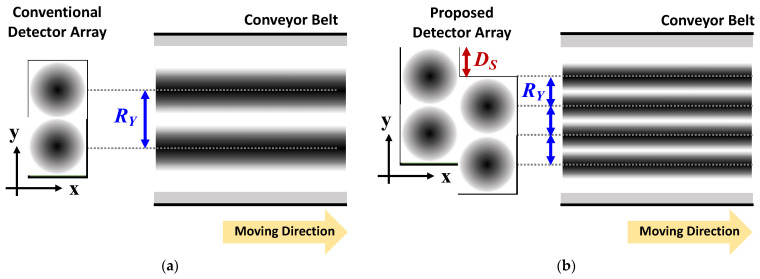
Operating principle to increase the spatial resolution of the proposed detector array: (**a**) Conventional detector array in which the detector unit pixels are linearly arranged. (**b**) Proposed detector array consisting of two columns of detectors that are staggered.

The proposed detector array increases the spatial resolution by using a configuration in which two or more detector columns are offset from one another. Assuming that the incident signals on adjacent detectors are similar owing to the rapid movement of the conveyor belt, the operating principle of the proposed method is based on the spatial resolution of the image obtained from the detector sub-array in the first column, which can be subdivided into the image obtained from the detector sub-array in the second column. Figure 2 presents the configuration of the proposed detector array. The detector unit pixels are composed of one detector IC individually mounted on a miniaturized board. The entire array structure is formed by attaching the unit pixels to at least two motherboard columns. To explain the spatial resolution improvement by the proposed array, it is assumed that the signal detected in the unit pixel has a Gaussian distribution with the center of the detector IC, which is described by the contrast of the circle shown in Figure 3, owing to the gain and beam width of the integrated antenna. Based on this assumption, a darker contrast area in Figure 3 indicates a more dominant effect on the detector output. As shown in Figure 3, the traveling direction of the conveyor belt is assumed to be along the x-axis, and the dimension for reducing the spatial resolution is along the y-axis. As shown in Figure 3a, the spatial resolution of the conventional array configuration, which places the detector unit pixel in a line, can be determined by the coverage of the unit pixel owing to the large gap between the pixels. The resolution limitation of the conventional configuration is caused by the unit pixel size, which is larger than the pixel coverage. Integrating several detectors on a single chip is a common method for overcoming this resolution limitation. In the proposed array shown in Figure 3b, the spatial resolution can be increased by using the second column, which is located between the pixels in the first column. This is based on the fact that incident signals at the same position perpendicular to the traveling direction of the conveyor belt are similarly detected by the pixels in the first and second columns.

When all the CMOS detectors in the array present voltage responses without failing or malfunctioning, the pixel pitch *R_Y_* on the y-axis of an image obtained by the conventional method is determined by the size of the detector unit pixel. Assuming that the same incident signal is applied to each detector between the different columns owing to the fast-moving speed of the conveyor belt, the pitch *R_Y_* can be reduced depending on the number of nonoverlapping columns in the proposed method, as shown in Figure 3b. The DC outputs of the conventional array, consisting of one column, can be expressed as an *n ×* 1 matrix *V_OC_* as follows:

(1) $$V_{OC}=\left[\begin{array}{c} \begin{array}{c} V_{o1}\\ V_{o2} \end{array}\\ \begin{array}{c} V_{o3}\\ \vdots \end{array}\\ \begin{array}{c} V_{o\left(n-1\right)}\\ V_{on} \end{array} \end{array}\right]$$

where *V_on_* is the DC output voltage of the *n^th^* unit detector in the array. Image resolution is defined as the number *n* of elements in the matrix. When the coverage length on the y-axis of the image obtained by the detector array is defined as *W_C_*, the obtained length *I_Y_*, which is directly connected to the image resolution, can be expressed as follows:

(2) IY=WCn .

In the proposed method consisting of two columns, the DC outputs can be expressed as a matrix *V_OP_* of 2*n* × 1 when the number of detectors in the first column is the same as that in the conventional method, and the second column is shifted to a distance of 0.5 × the y-direction length of the unit pixel as follows:

(3) $$V_{OP}=\left[\begin{array}{c} \begin{array}{c} V_{o1}\\ 0.5\left(V_{o1}+V_{o2}\right) \end{array}\\ \begin{array}{c} 0.5\left(V_{o2}+V_{o3}\right)\\ \vdots \end{array}\\ \begin{array}{c} 0.5\left(V_{o\left(n-1\right)}+V_{on}\right)\\ V_{on} \end{array} \end{array}\right]$$

In the proposed array, the image resolution increased to *2n*, and the pixel pitch of the image is reduced by 1/2 times the length *I_Y_*. Proportionally increasing the image resolution with the number of columns can be implemented by positioning each column with the shifting distance *D_S_*, which is calculated as follows:

(4) DS=LYk ,

where *L_Y_* denotes the length of the unit pixel in the y-direction, and *k* denotes the number of columns in the proposed array. 

## 3. Implementation of the Proposed CMOS Detector Array 

### 3.1. CMOS Detector Integrated Circuit

**Figure 4:**
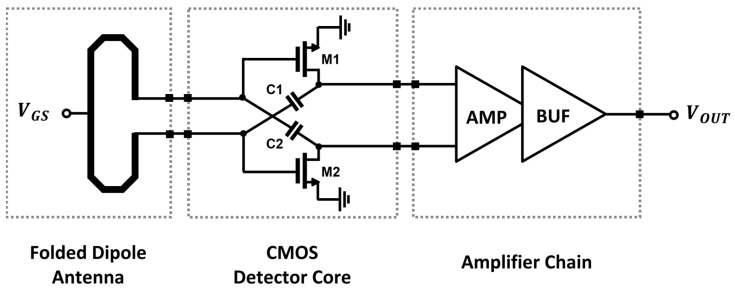
Block diagram of the CMOS detector-integrated circuit for detector unit pixel of the proposed array.

A CMOS detector IC is designed by using a detector core with a concurrent mode topology to increase the voltage responsivity (*R_V_*) to the incident signal [26]. The detector unit pixel consists of a folded dipole on-chip antenna, a preamplifier combining the DC outputs from the differential detector core transistors, and a unit-gain voltage buffer, as shown in Figure 4 [27,28]. 

#### 3.1.1. Folded Dipole on-Chip Antenna

**Figure 5:**
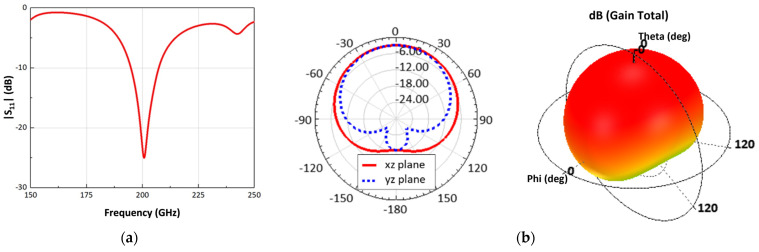
Three-dimensional electromagnetic simulation results of the folded dipole on-chip antenna: (**a**) Reflection coefficient. (**b**) Radiation pattern for indicating the gain and 3-dB beamwidth.

A folded dipole antenna is designed on a chip to implement a topology that requires differential inputs and gate biasing from a common node. A 3D EM simulation was performed to design the antenna by using the top metal layer of backend oxide layers (BEOL) provided by the CMOS process as the radiation and the bottom of the silicon substrate as the ground plane. The guard ring structure using all the metal layers in the BEOL surrounds the antenna to diminish the coupling effect with adjacent detector pixels. The space between the guard ring structure and the antenna radiating metal is sufficiently spaced at a quarter wavelength to reduce the effect of grounding on the efficient spatial radiation. The antenna has an operating frequency of 200 GHz and is 0.5 × 0.2 mm^2^ in size, which is based on the area determined by the guard ring. The operating frequencies of the antenna, based on the magnitude of *S_11_* of −10 dB or less, were simulated from 194.8 to 207 GHz, as shown in Figure 5a. Figure 5b demonstrates that the simulated antenna gain and 3-dB beamwidth are to be −2.95 dBi and 104°, respectively.

#### 3.1.2. Differential Detector Core

**Figure 6:**
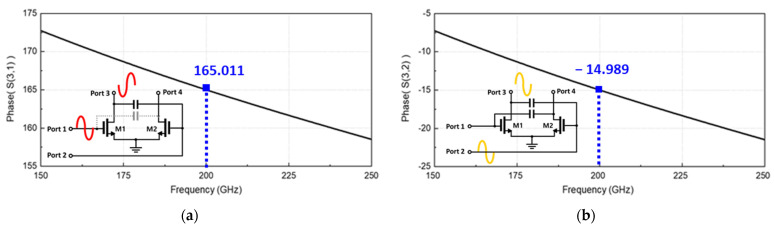
Simulated phase difference to the output node of the detector core transistor from: (**a**) a positive input port and (**b**) negative input port.

**Figure 7:**
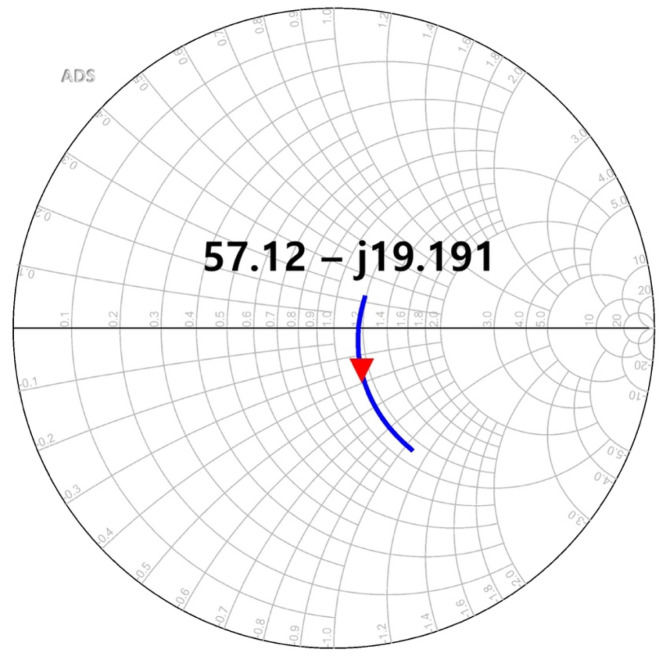
Simulated input impedance at a single-ended port of the detector core.

The concurrent-mode topology can obtain the maximum *R_V_* by the in-phase transmission of the differential input signals transmitted to the output drain node of the detector core transistor [26]. The differential incident signals are simultaneously input to the gate and drain nodes of the single detector core transistor and are individually generated to DC outputs at the drain node. An in-phase signal combination for increasing the detection output can be generated by implementing a phase shift of 180° by using a cross-coupled line and an additional capacitor. In the proposed detector core, the in-phase transmission was achieved using a cross-coupled line with a length of 18.6 µm and an additional capacitance of 58.5 fF, as shown in Figure 6. The phase from the positive input port to the output of the detector core transistor shown in Figure 6a and that from the negative port to the same output shown in Figure 6b demonstrated a difference of 180° by using the EM simulation. Impedance matching between the antenna and detector core was obtained using a transistor that was 1.2 µm × 65 nm (gate width × gate length), and the length of the transmission line was 90.6 µm, connecting the antenna and the detector core, as shown in Figure 7.

#### 3.1.3. Amplifier Chain

**Figure 8:**
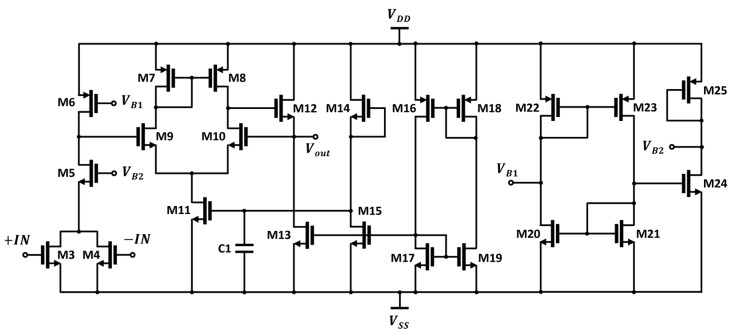
Schematic of the preamplifier and voltage buffer in the CMOS detector IC for the proposed detector array.

Figure 8 presents a schematic of the differential to the single-ended amplifier chain, consisting of a preamplifier and voltage buffer with bias circuitry. The DC voltage output from the differential detector core is applied to transistors M3 and M4, which are low threshold voltage devices and converted into a current signal passing through M5 and M6. The leakage signals in the sub-terahertz band are canceled at the M3 and M4 drain nodes and are not delivered to the output of the preamplifier [27]. The bias voltages *V_B1_* and *V_B2_* of the preamplifier were designed to be 0.65 V and 0.4 V, respectively, to have the maximum voltage responsivity at the output by bias circuit-implemented diode-connected transistors and current mirrors. A voltage buffer configured with a source follower provides feedback to increase both the *R_V_* and stability [26]. The bias circuits, consisting of the diode connection and current mirrors, are implemented with transistors M14 to M18 for a robust design for process variation.

### 3.2. Chip Fabrication and Unit Detector Module

**Figure 9:**
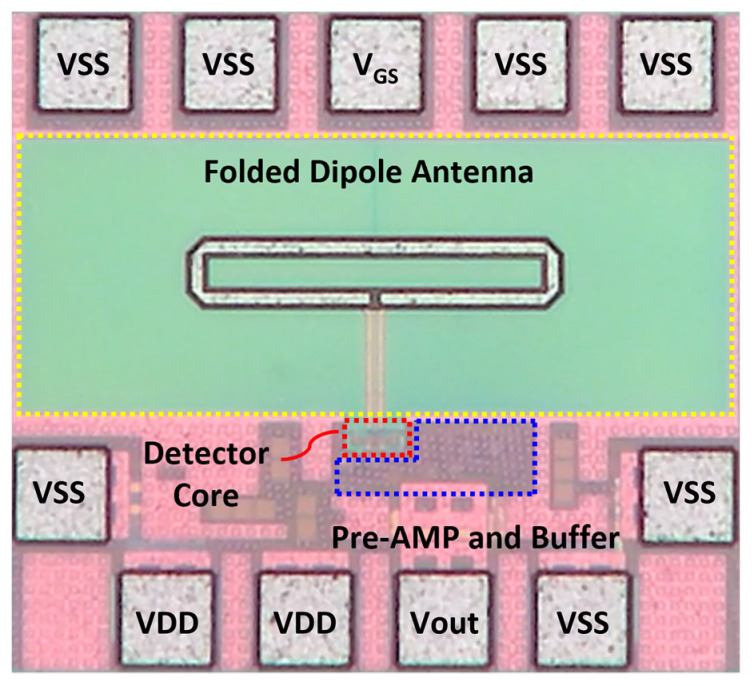
Photograph of the CMOS detector IC for the unit pixel of the proposed array fabricated on the 65 nm CMOS process.

**Figure 10:**
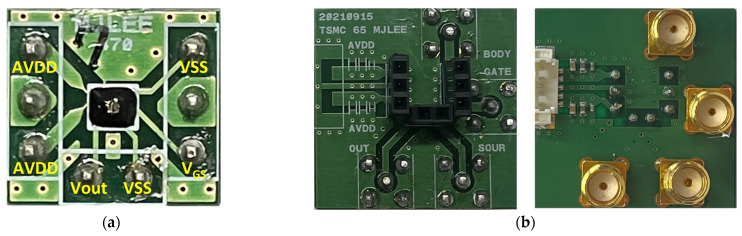
(**a**) Photograph of the CMOS detector unit module. (**b**) Adapter board to measure the voltage responsivity and noise-equivalent power of the unit module.

An integrated antenna, differential detector cores, and an amplifier chain were fabricated into a single chip detector using the 65-nm 1-poly 9-metal CMOS process, as shown in Figure 9. Considering the free space that does not affect the antenna radiation characteristics, the chip area was implemented on 500 × 450 µm^2^, including the wire-bonding pads. The DC bias pads for supplying the ground and the gate voltage of the detector core are located at the upper part of the fabricated IC, as shown in Figure 9, and the output signal and other bias pads of the detector IC are located at the lower part for a simple interface of the detector array. As shown in Figure 10a, the detector unit module using the fabricated IC was implemented on a PCB that was 1.08 cm × 1 cm (width × length). All the pads shown in Figure 9 were connected to each terminal through bonding wires. The integrated antenna, located at the center of the detector IC, is not covered by epoxy and was directly exposed to the input signal. Figure 10b demonstrates the adapter board used to measure the detector performance of the unit module. The unit module and adapter board are connected through detachable pin array sockets. Using the adapter board helps the proposed detector array to be configured with unit modules which operating characteristics have been verified from the measurement results of the *R_V_*.

### 3.3. CMOS Detector Array

**Figure 11:**
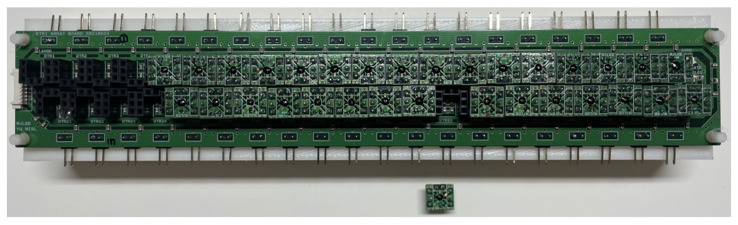
Photograph of the proposed 16 × 2 detector array structure with two columns.

Each detector unit module was connected to the motherboard, which is 240 mm × 30 mm, as shown in Figure 11, to form the proposed 16 × 2 detector array. A low-quality unit module, which has low detection characteristics and malfunctions, can be replaced in the proposed array, because the interface is implemented with the same pin array sockets used in the adapter board. All bias voltages supplied to the unit modules are equally applied, and the effect of the voltage drop of the interconnection lines is minimized by a parallel connection. The output of the unit module in the proposed array is individually configured for connection to the data acquisition board with multi-channel inputs. When the assumptions of the conveyor belt speed and incident signal on each column of the detector array indicated in Section 2 are valid, the proposed array structure can increase the image resolution by increasing the number of columns. Increasing the number of columns can increase the complexity of implementing the system, as both a faster-moving speed of the conveyor belt and a higher sampling rate for acquiring the data are required. This assumption can become invalid, as the differences in the sampling times between the unit detectors increase. The columns used in this study are set to two lines to demonstrate the effectiveness of the proposed array.

## 4. Measurement Results and Discussion

### 4.1. Voltage Responsivities of CMOS Detector Unit Modules

**Figure 12:**
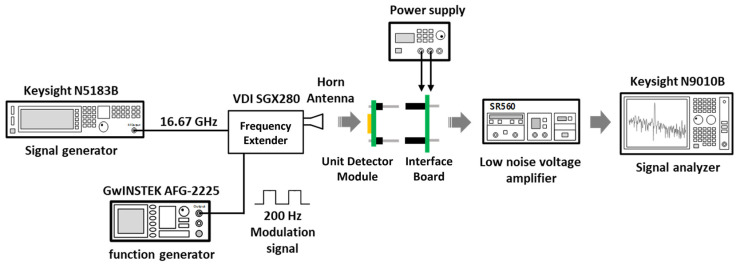
Experimental setup for measuring the voltage responsivity of the detector unit module.

**Figure 13:**
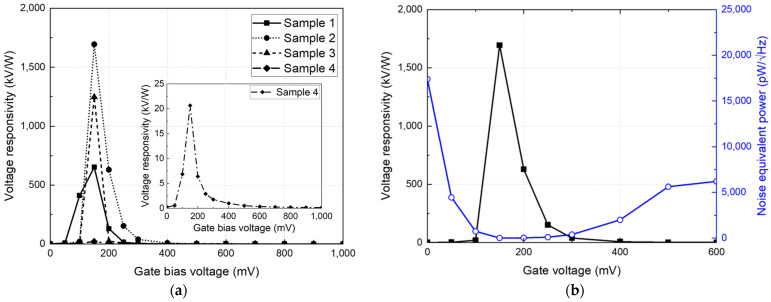
Detection performances of the CMOS detector unit module: (**a**) Voltage responsivities of four detector unit modules randomly selected in the array. (**b**) Voltage responsivity and noise-equivalent power of sample 2 among the modules.

Figure 12 presents the measurement setup for the CMOS detector unit module. A 0.2 THz signal is generated using a fundamental frequency of 16.7 GHz and a frequency multiplication factor of 12. A frequency modulation of 200 Hz is input to the frequency multiplier to reduce the deterioration of the detection characteristics owing to the flicker noise. The detector output is measured at 200 Hz by modulation, and the *R_V_* is calculated from the output variation, which depends on the presence or absence of the input signal and the incident power at the distance of the detector module [29]. The incident power is obtained from the effective antenna area calculated from the antenna gain and the measured power at the distance where the detector is placed by using the signal analyzer. The module is aligned to the optimum position, where the output variation is maximized using an X–Y moving stage before obtaining the output of the module. An external low-noise amplifier is used to provide the voltage gain and passband of the modulation frequency, and its gain is compensated for in the calculation of the voltage responsivity. The *R_V_* obtained for each of the four detector modules are shown in Figure 13a. The maximum *R_V_* of the unit modules were measured as 651 kV/W, 1.694 MV/W, 1.247 MV/W, and 20.6 kV/W, respectively, at the optimum bias voltage of 150 mV. The different characteristics of *R_V_* indicate that the normalization process is essential in a detector array composed of detector unit modules. The noise equivalent power (*NEP*) for sample 2, which had the highest *R_V_* among the four samples shown in Figure 13a, was 9 pW/√Hz at a gate bias of 150 mV, as shown in Figure 13b. Table 1 shows a performance comparison of the 200 GHz CMOS detectors. The CMOS detector unit module shows lower *R_V_* due to the low voltage gain of the integrated amplifier chain and low supply voltage of the 65 nm CMOS process compared to previous studies using the same configuration of the detector core. However, the *NEP* of the detector module was improved more than four times compared to previous detectors manufactured by the 250 nm process. This performance improvement means that the module can provide high image quality with low minimum sensitivity in the sub-terahertz imaging system.

### 4.2. Images of the Target on the Conveyor Belt

**Figure 14:**
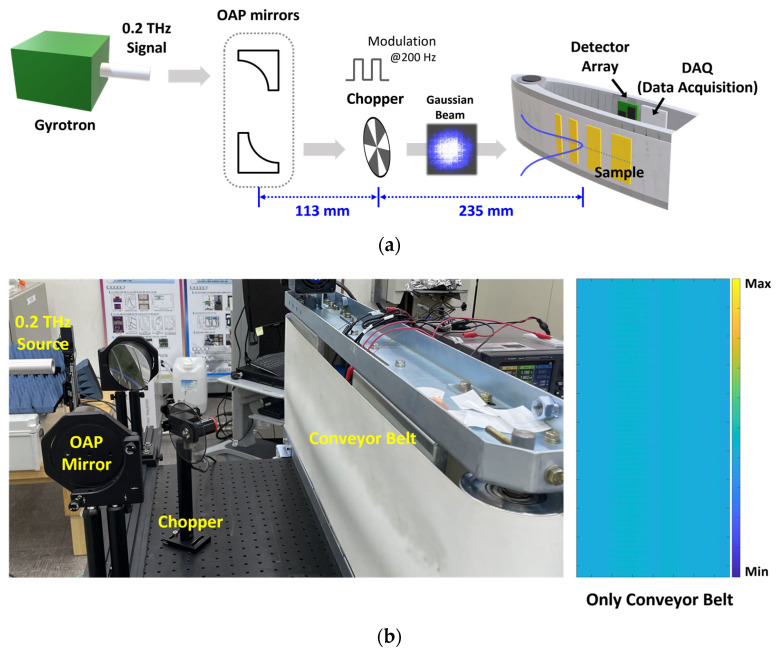
Experimental setup for measuring the sub-terahertz images using the proposed CMOS detector array: (**a**) Block diagram of the setup. (**b**) Photo of the setup and the measured image when there is no object on the conveyor belt.

The experimental setup for the target object on the conveyor belt is shown in Figure 14. The 0.2 THz signal generated by the gyrotron is applied to the object on the conveyor belt through off-axis parabolic (OAP) mirrors [24]. Instead of electrical modulation for the measurement of the unit module, a mechanical chopper modulates the incident signal with a frequency of 200 Hz to be less affected by the flicker noise of the detector core transistors. The proposed detector array is placed inside the conveyor belt, and a data acquisition board with 32 input channels is used to obtain the output of the array. The incident signal is spread to the conveyor belt positioned 235 mm distant from the chopper; however, the signal is in the form of a spot with a Gaussian beam profile and cannot expose the entire shape of the object on the conveyor belt. The spot beam profile of the incident signal is confirmed in the image measured without placing any object on the conveyor belt, as shown in the center of Figure 14a. A high response output is generated in the center of the belt, where the incident signal power was high, and the outputs in the array decrease toward the outer edge of the belt owing to the beam distribution of the incident signal. The initial image in Figure 14b is used to compensate for the attenuation and scattering of the incident signal by the belt and the beam profile characteristics when the detector has a sufficient dynamic range to describe the intensity of the incident signal. The moving speed of the object was set to 16 mm/s by the conveyor belt, which was the maximum speed at which unwanted vibrations did not occur in the measurement environment.

**Figure 15:**
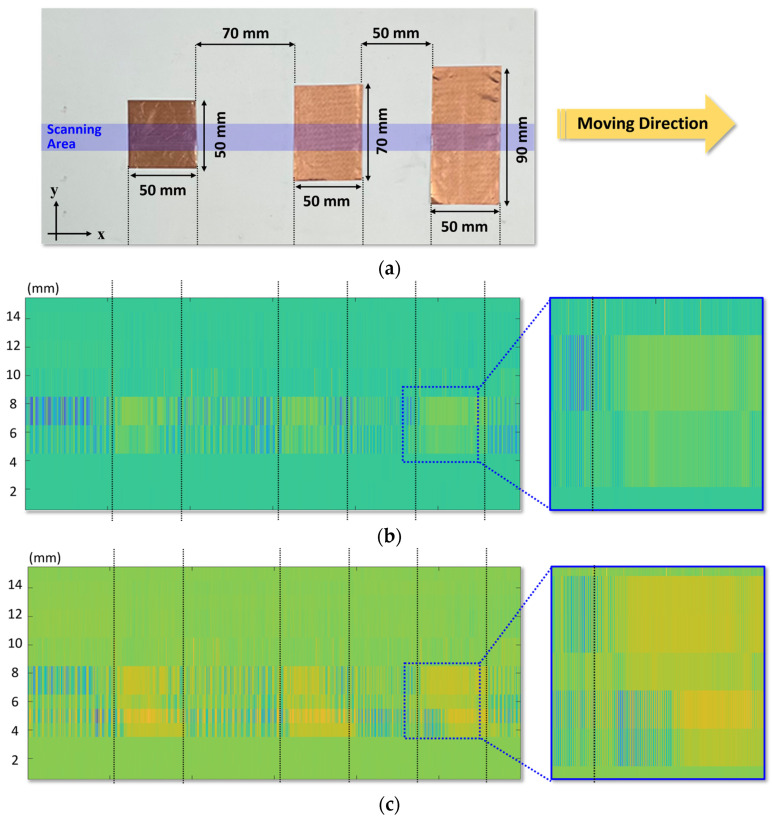
Measured sub-terahertz images: (**a**) Photo of the target object on the conveyor belt with the physical dimension of the object. (**b**) Image obtained by using the conventional array with one column consisting of 16 modules. (**c**) Image obtained by using the proposed array with two columns consisting of 16 × 2 modules.

Figure 15a presents the target object placed on the conveyor belt. Patterns composed of copper foil tapes with a thickness of 60 µm were placed at intervals of 70 mm and 50 mm, and the spot beam profile of the incident signal obtained from the initially obtained image is shown in blue in Figure 15a. Figure 15b,c present the sub-terahertz images obtained from the conventional and proposed array structures. Both images demonstrate that the objects on the belt can be distinguished and were obtained by the voltage difference of the detector output exhibited by the incident signal masked by the copper tape. The image resolution obtained from the conventional array consisting of one column was displayed at 10 mm, which was determined from the intervals of the detector array configuration, as shown in Figure 15b. Based on the images obtained from the proposed array, the image resolution was enhanced to 5 mm, as shown in Figure 15c. The SNR in the images, which is defined as the ratio of the maximum and minimum voltages in each image, was calculated to be 12.1 dB in the proposed array. Despite the low conveyor belt speed of 16 mm/s, the improvement in the image resolution by the proposed array demonstrates that the proposed array can provide a high-resolution image when sub-terahertz signals with a linear beam profile are uniformly irradiated to the target object on the conveyor belt.

## 5. Conclusions

A CMOS detector array structure is proposed to increase the image resolution of a moving object on a conveyor belt. In arranging an additional column of detectors perpendicular to the moving direction of the conveyor belt, the detectors in the second column of the proposed array are staggered in the middle of those in the first column. When the conveyor belt is assumed to move sufficiently fast and the sub-terahertz signal is incident on the adjacent detectors with nearly the same intensity, the image resolution in the vertical direction of the movement of the belt can be increased by the proposed array, which can be configured by attaching detector unit modules to any position on the motherboard. The unit module can replace the motherboard when the detection performance of the module is abnormal. The CMOS detector IC, consisting of a folded dipole on-chip antenna, a concurrent-mode detector core, and an amplifier chain with a preamplifier and voltage buffer, was fabricated using a 65 nm CMOS process. The detector IC for the unit module exhibited a voltage responsivity of 1.69 MV/W and noise-equivalent power of 9 pW/√Hz for an incident signal of 0.2 THz. The proposed array structure implemented as a 16 × 2 array configuration doubled the vertical image resolution of an object placed on the conveyor belt moving at 1.6 mm/s. In addition, it can increase the vertical image resolution perpendicular to the moving direction of the conveyor belt, by the number of additionally arranged columns, which can be useful for improving the quality of real-time nondestructive images for foreign body detection.

## Figures and Tables

**Table 1:** Performance comparison of the 200 GHz CMOS detector-integrated circuits.

Ref.	Process (nm)	Freq. (GHz)	Detector Core Configuration	*R_V_* (kV/W)	*NEP* (pW/√Hz)
[26]	250	200	Concurrent mode	14,130	34.4
[27]	250	200	Gate-coupled	2020	76
[28]	250	200	Gate-coupled	357	57.3
[29]	250	200	Gate-coupled	482	39.3
[30]	250	200	Gate-coupled	5696	62.4
[31]	250	200	Gate-coupled	2990	46.3
[32]	350	200	Gate-coupled	19	535
[33]	65	300	Source-coupled	3.6	12.5
[34]	65	310	Drain-coupled	2.0	3.5
This works	65	200	Concurrent mode	1694	9.0

## Data Availability

The data presented in this study are available on request from the corresponding authors. The data are not publicly available due to the regulations of the project.

## References

[1] Vasquez J.T., Scapaticci R., Turvani G., Ricci M., Farina L., Litman A., Casu M.R., Crocco L., Vipiana F. (2020). Noninvasive inline food inspection via microwave imaging technology: An application example in the food industry. IEEE Antenna Propag. Mag..

[2] Ahmed S.S. (2021). Microwave imaging in security—Two decades of innovation. IEEE J. Microw..

[3] Deng Y., Liu X. (2011). Electromagnetic imaging methods for nondestructive evaluation applications. Sensors.

[4] Mehrotra P., Chatterjee B., Sen S. (2019). EM-wave biosensors: A review of RF, microwave, mm-wave and optical sensing. Sensors.

[5] Simonov N., Jeon S.-I., Kim B.-R., Son S.-H. (2018). Analysis of the super-resolution effect on microwave tomography. Radio Sci..

[6] Sun X., Li J., Shen Y., Li W. (2021). Non-destructive detection of insect foreign bodies in finishing tea product based on terahertz spectrum and image. Front. Nutr..

[7] Yang J.-R., Lee W.-J., Han S.-T. (2016). Signal-conditioning block of a 1 × 200 CMOS detector array for a terahertz real-time imaging system. Sensors.

[8] Stec B., Susek W. (2018). Theory and measurement of signal-to-noise ratio in continuous-wave noise radar. Sensors.

[9] Ok G., Park K., Chun H.S., Chang H.-J., Lee N., Choi S.-W. (2015). High-performance sub-terahertz transmission imaging system for food inspection. Biomed. Opt. Express.

[10] Ding S.-H., Li Q., Yao R., Wang Q. (2010). High-resolution terahertz reflective imaging and image restoration. Appl. Opt..

[11] Zhao J., Chu W., Wang Z., Yang J., Liu W., Chung Y., Xu Z. (2014). Terahertz imaging with sub-wavelength resolution by femtosecond laser filament in air. Sci. Rep..

[12] Liu Z.-Y., Qi F., Wang Y.-L., Liu P.-X., Li W.-F. A 150-to1050 GHz terahertz detector in 65 nm CMOS. Proceedings of the IEEE Asian Solid-State Circuits Conference (A-SSCC).

[13] Balow A.M., Khatir M., Amiri N. (2021). Terahertz detection using large-area plasmonic nano-antenna arrays based on stepped strips. Optik.

[14] Zhao F., Mao L., Guo W., Xi S., Tee C.A.T.H. (2020). On-chip terahertz detector designed with inset-feed rectangular patch antenna and catadioptric lens. Electronics.

[15] Lee C., Jeong J. (2020). THz CMOS on-chip antenna array using defected ground structure. Electronics.

[16] Liao Y., Wang K., Zhu H., Ji X. (2022). Crosstalk in CMOS terahertz detector array with on-chip SPR antenna. IEEE Photonics J..

[17] Pham H.H.N., Hisatake S., Minin O.V., Nagatsuma T., Minin I.V. (2017). Enhancement of spatial resolution of terahertz imaging systems based on terajet generation by dielectric cube. APL Photonics.

[18] Mostajeran A., Aghasi H., Naghavi S.M.H., Afshari E. Fully integrated solutions for high resolution terahertz imaging. Proceedings of the IEEE Custom Integrated Circuits Conference (CICC).

[19] Hu Z., Wang C., Han R. (2019). A 32-unit 240-GHz heterodyne receiver array in 65-nm CMOS with array-wide phase locking. IEEE J. Solid-State Circuits.

[20] Xu L.-M., Fan W.-H., Liu J. (2014). High-resolution reconstruction for terahertz imaging. Appl. Opt..

[21] Ahi K. (2019). A method and system for enhancing the resolution of terahertz imaging. Measurement.

[22] Lei T., Tobin B., Liu Z., Yang S.-Y., Sun D.-W. (2021). A terahertz time-domain super-resolution imaging method using a local-pixel graph neural network for biological products. Anal. Chim. Acta.

[23] Lee Y.-K., Choi S.-W., Han S.-T., Woo D.H., Chun H.S. (2012). Detection of foreign bodies in foods using continuous wave terahertz imaging. J. Food Prot..

[24] Han S.-T. (2020). Application of a compact sub-terahertz gyrotron for non-destructive inspections. IEEE Trans. Plasma Sci..

[25] Rangan S., Rappaport T.S., Erkip E. (2014). Millimeter-wave cellular wireless network: Potentials and challenges. Proceeding IEEE.

[26] Lee M.-J., Lee H.-N., Lee G.-E., Han S.-T., Yang J.-R. (2022). Concurrent-mode CMOS detector IC for sub-terahertz imaging system. Sensors.

[27] Yang J.-R., Han S.-T., Baek D.-H. (2017). Differential CMOS sub-terahertz detector with subthreshold amplifier. Sensors.

[28] Lee H.-N., Lee H.-J., Han S.-T., Yang J.-R. (2021). Highly sensitive CMOS plasmon detector with a low-gain buffer amplifier for terahertz imaging system. Microw. Opt. Technol. Lett..

[29] Son J.-H., Yang J.-R. (2019). Quasi-static analysis based on an equivalent circuit model for a CMOS terahertz plasmon detector in the subthreshold region. Sensors.

[30] Lee H.-J., Han S.-T., Yang J.-R. (2020). CMOS plasmon detector with three different body-biasing MOSFETs. IEEE Access.

[31] Lee G.-E., Lee H.-J., Han S.-T., Yang J.-R. (2021). CMOS detector using customized bolt-wrench capacitor on backend oxide layer. IEEE Microw. Wirel. Compon. Lett..

[32] Hassanalieragh M., Ignjatovic Z., Newman J.D., Fourspring K. (2020). Design and characterization of a 10 × 10 pixel array THz camera in 350 nm CMOS technology. IEEE Sens. J..

[33] Song K., Kim J., Kim D., Yoo J., Rieh J.-S. (2020). A 300-GHz CMOS 7-by-7 detector array for optics-less THz imaging with scan-less target object. J. Infrared Millim. Terahertz Waves.

[34] Shaulov E., Jameson S., Socher E. (2021). A zero bias J-band antenna-coupled detector in 65-nm CMOS. IEEE Trans. Terahertz Sci. Technol..

